# Cholecystitis1Read at the Thirty-sixth Annual Meeting of the Medical Association of Central New York, at Buffalo, October, 1905.

**Published:** 1906-02

**Authors:** DeLancey Rochester

**Affiliations:** Buffalo, N. Y.. Associate Protessor of Principles and Practice of Medicine, University of Buffalo


### Cholecystitis[1]

By DeLANCEY ROCHESTER, M. D., Buffalo, N. Y..
Associate Protessor of Principles and Practice of Medicine, University of Buffalo.

"CHOLESTERIN," says Mayo Robson, "which is the main constituent of a biliary calculus, is derived, not, as used to be thought, from the bile, but from the epithelial lining of the gall bladder or bile ducts.......... It seems probable, therefore, that the origin of gallstones is to be sought, not in any alteration in the characters or rate of flow of the bile, but rather in the local condition of the mucous membrane of the bile passages. During the last twenty years from all sides evidence has been accumulating which tends to show that this preliminary condition consists of a bacterial infection of the bile channels from the intestine. Normally bile is sterile, but in practically all cases of cholelithiasis, microorganisms can be found if carefully looked for." Later, in the same article he says, "It should be remembered that gallstones are probably invariably present before primary malignant disease of the gall bladder or bile ducts." These statements of Robson can, I think, be accepted as true. Thus we see that bacterial invasion is the precursor of all serious diseases of the gall bladder and bile ducts. The organism most frequently found present in such cases has been the colon bacillus, next the typhoid bacillus, and in small numbers, about equally distributed, the influenza bacillus, the pneumococcus, the stapylococcus albus and aurens and the streptococcus, and the cholera bacillus.

An infectious cholecystitus, its etiology, its diagnosis, its treatment and its prevention, is the subject to which I wish to direct your attention.

From the study of cases of acute cholecystitis associated with typhoid fever which have come to autopsy, it is demonstratable that inflammation extends from the duodenum. In other acute inflammatory states, such as grippe, etc., it is fair to assume the same route of invasion has been followed. In some cases of subacute or chronic inflammation the route is undoubtedly the same as in the acute form referred to; in other forms, especially, those of infection by the colon bacillus, it is quite probable that the route of invasion has been through lymphatic channels or through the portal circulation and possibly in some cases by migration from hepatic flexure of colon direct to gall bladder.

From what has been said it is plain that the chief etiologic factor in cholecystitis is a preceeding inflammatory condition of

1. Read at the Thirty-sixth Annual Meeting of the Medical Association of Central New York, at Buffalo, October, 1905.

the gastric-intestinal tract, especially of the duodenum and of the colon. Dseases of lung or heart which tend to produce portal congestion, derangements of gastric, enteric or colonic secretions from any cause, are thus seen to enter into the etiology of this disease. These disturbances of the digestive tract act in two ways as etiologic factors; in the first place as source of extension of inflammation and in the second place as reducing the general nutrition and so reducing the resisting power of the individual to infection.

While in a large number of cases gallstones are present in the gall bladder without producing any symptoms, sometimes the presence of gall stones keeps up an inflammation and sometimes increases it and even after gall stones have been removed, either by passage into intestine or by operative procedure, cholecystitis sometimes persists.

Three forms of acute cholecystitis are described, the catarrhal, the suppurative and the phlegmonous; they really are grades of inflammation rather than distinct varieties.

The gall bladder is distended, the lumen of the cystic duct narrowed or closed through swelling of the mucosa or plugged by mucus or gall stone—adhesions may have formed with the omentum or the colon or perforation may have occurred, resulting in local peritonitis and abscess or in general peritonitis. The contents of the gall bladder are usually dark in color and may be mucopurlent, purulent or hemorrhagic in character and in some cases of very foul odor. Experimental infection of the gall bladder has been made with streptococcus and with colon bacillus and no symptom occurred if the outflow of bile was not interferred with. For the recognition of cholecystitis by symptoms and signs it is necessary that there should be some obstruction of the cystic or common bile duct, interferring with the outflow of the bile from the gall bladder. There must, therefore, be swelling of the mucosa of the cystic duct or of the common duct sufficient to obstruct its lumen or there must be external tumor producing pressure or foreign body, gall stone, or inspissated mucus in the lumen of one or the other duct.

The course of a cholecystitis occurring as a complication of an infectious fever—especially typhoid—is a little different from the course of a case arising in association with more chronic disturbance of the intestinal tract. In the four cases of cholecystitis associated with typhoid which I have seen, the onset has been rather abrupt, with nausea, in two cases vomiting, pain or feelng of distress or fulness in the epigastrum or, in one case, in the right hypochondrium: there has always been an increase in the temperature and in the frequency of the pulse; in two cases there was a moderate leucocytosis, in two none; in all, the area of hepatic dullness was increased, there was rigidity of the right rectus, tenderness and a palpable tumor extending toward the umbilicus from the tip of the ninth rib. In none of them was jaundice present.

In the acute infectious cases, associated with cholangitis, jaundice is invariably present, developing rapidly with the chill, nausea and vomiting that are present. In all acute cases, not of typhoid origin, and in some due to typhoid, leucytosis is present. In cases associated with gall stones we have the history of the hepatic colic and we may or may not have jaundice.

In the violently acute, fulminating cases we have greater violence of pain, more pronounced vomiting, cold sweat and collapse, together with general abdominal rigidity and tenderness, though by careful manipulation a point of greatest tenderness can be generally discovered in the region of the gall bladder and relative percussion dullness can be elicited even when a tumor cannot be felt on account of rigidity of abdominal walls.

The diagnosis of acute cholecystitis will depend upon a history of present existing infectious fever or other morbid state producing an inflammation of the duodenum or a history of gall stones; upon suddenness of onset with chill, nausea, vomiting, pain in right hypochondrium or epigastrium, rigidity of abdominal wall, tumor demonstrable by palpation or percussion and upon leucocytosis or Widal reaction or both. The most constant sign in all forms of the disease is tenderness to pressure in the region of the gall bladder. Even in cases where tumor cannot be satisfactorily demonstrated, this tenderness may be elicited when the examiner's fingers are pressed up under the ninth costal cartilage and the patient attempts to take a full breath. In cases of moderate intensity the tenderness is relatively slight, but there is produced spasmodic rigidity of the abdominal wall. In cases of greater violence, the tenderness is pronounced, there occurs spasms of the diaphragm and the patient's attempt to take a full breath is cut off short. The presence of this tenderness in all forms of cholecystitis is especially noted by Murphy. (Medical News, 1904, p. 825.)

In most cases the diagnosis is not difficult though there are some in which it is impossible without laparotomy. The rapidly developing cases of the phlegmonous type are the most difficult. The two diseases which they most resemble are acute appendicitis and intestinal obstruction.

As regards the former, the site of the tenderness and of the tumor together with the previous history of the case are important. We must not forget that the two diseases may coexist. I

have seen one such case in consultation with Dr. Stockton. In intestinal obstruction it is uncommon to find the tumor in the neighborhood of the gall bladder. The previous history of the case will usually help also.

As regards the treatment of cholecystitis, while two of my cases occurring with typhoid fever recovered without operation and while autopsies reveal the fact that gall stones may be present in the gall bladder for years and produce no symptoms, nevertheless I am of the opinion that the best advice we can give a patient suffering with cholecystitis is to have the gall bladder drained by surgical procedure. The gall bladder may be irrigated and drainage continued until the contents comes away sterile when the wound may be closed.

In cases where it can be done without too much injury to surrounding structure, it would probably be best to remove the gall bladder entirely. In cases of the rapidly developing acute phlegmonous or suppurating forms, operative procedure is imperative. In cases occurring with gall stones, operation should be urged. In mildly acute catarrhal forms of the disease, the necessity for operation seems to the patient rather remote. Under such circumstances and in those cases associated with stone, who decline operation, what is to be done?

I wish to state briefly here why I think it wise to have all cases of cholecystitis operated. Because I believe cholecystitis is infectious, infection of the gall bladder may subside possibly, but probably does not and is liable to become violently acute, producing local peritonitis and so obstruction of bowel or empyema of gall bladder, conditions which require operation for recovery; or, if these violent conditions do not supervene, this infecting organism it apt to produce a subacute or chronic catarrhal state and become the nidus for stone and so possibly for malignant disease.

The particular operation recommended would depend upon the especial conditions present in a given case. If, however, operation is declined, and the acute symptoms subside, is there any line of treatment which would be of benifit in tending to diminish the tendency to formation of stone and perhaps restore the normal condition of the gall bladder?

In those forms, not associated with stone, occurring in the course of typhoid fever, the treatment should first be directed toward alleviating the pain and discomfort, nausea, etc. of the patient. For this purpose the prompt application of from four to six leeches over the tender area and along the free border of the ribs, followed by a light ice bag, renewed frequently enough to keep it cold, may be all that is necessary. Usually, however, in addition to these procedures a hypodermic injection of morphine in full dose is required. After this, the regular daily use of a moderate dose of carlsbad salts each morning and the use of large amounts of distilled water rendered mildly alkaline by sodium bicarbonate and the restriction of the diet to beef juice, raw white of egg and strained meat broths, entirely freed from grease, until the acute symptoms have subsided, give the best results. The persistent use of sodium salicylate in small doses frequently repeated seems to have been of distinct benefit in some cases. Experimentally, the salicylates have apparently rendered previously infected bile vessels aseptic. If so, it is a valuable drug in the treatment of this condition and for use after operation for gall stones.

When cholecystitis is present associated with gall stones, the rational procedure is of course removal of the stones.

I have never been convinced that by the administration of any medicine, gall stones already formed can be dissolved. A great deal, however, can be done to put the intestinal canal in good shape and so tend to keep the bile ducts patulous and possibly the persistent use of salicylic acid or some of its salts may tend to render the bile passages, including the gall bladder, aseptic.

Much can be done, also, to render attackes of biliary colic less frequent and less severe by so adjusting clothing that pressure by belts or corsets over the liver and region of the gall bladder is prevented; by regulating diet so that bland food, in the minimum amount compatable with health, is taken, that plenty of mildly alkaline water is used; that the bowels are kept free by the regular use of carlsbad, epsom or Glauber salts; that the colon is kept clean by hot enemata of boric acid solution taken two or three times a week and general metabolism kept at par by regular systematic exercises taken out of doors.

By such measures persistently kept up, cases which have had several attacks of biliary colic, may be kept free from recurrence.

If, however, in spite of such procedure, the attacks recur, I think we should be greatly remiss in our duty to our patient if we did not insist upon operation.

469 Franklin Street.

